# microRNAs and Acute Myeloid Leukemia Chemoresistance: A Mechanistic Overview

**DOI:** 10.3389/fonc.2017.00255

**Published:** 2017-10-30

**Authors:** Martino Marco Gabra, Leonardo Salmena

**Affiliations:** ^1^Department of Pharmacology and Toxicology, University of Toronto, Toronto, ON, Canada; ^2^Princess Margaret Cancer Centre, University Health Network, Toronto, ON, Canada

**Keywords:** microRNA, acute myeloid leukemia, drug resistance, RNA therapy, daunorubicin, cytarabine, chemotherapy

## Abstract

Up until the early 2000s, a functional role for microRNAs (miRNAs) was yet to be elucidated. With the advent of increasingly high-throughput and precise RNA-sequencing techniques within the last two decades, it has become well established that miRNAs can regulate almost all cellular processes through their ability to post-transcriptionally regulate a majority of protein-coding genes and countless other non-coding genes. In cancer, miRNAs have been demonstrated to play critical roles by modifying or controlling all major hallmarks including cell division, self-renewal, invasion, and DNA damage among others. Before the introduction of anthracyclines and cytarabine in the 1960s, acute myeloid leukemia (AML) was considered a fatal disease. In decades since, prognosis has improved substantially; however, long-term survival with AML remains poor. Resistance to chemotherapy, whether it is present at diagnosis or induced during treatment is a major therapeutic challenge in the treatment of this disease. Certain mechanisms such as DNA damage response and drug targeting, cell cycling, cell death, and drug trafficking pathways have been shown to be further dysregulated in treatment resistant cancers. miRNAs playing key roles in the emergence of these drug resistance phenotypes have recently emerged and replacement or inhibition of these miRNAs may be a viable treatment option. Herein, we describe the roles miRNAs can play in drug resistant AML and we describe miRNA-transcript interactions found within other cancer states which may be present within drug resistant AML. We describe the mechanisms of action of these miRNAs and how they can contribute to a poor overall survival and outcome as well. With the precision of miRNA mimic- or antagomir-based therapies, miRNAs provide an avenue for exquisite targeting in the therapy of drug resistant cancers.

## Introduction

Despite rapid progress in our understanding of the cellular and molecular etiology of cancer and the development of countless new anticancer agents and therapeutic strategies, little has changed in the treatment of many cancers over the last few decades. For instance, the standard of care for acute myeloid leukemia (AML) which consists of combined cytarabine and anthracycline therapy has been fundamentally unchanged for the past 30 years (1). The long-standing presence of this strategy is owed to its effectiveness with a mean response rate up to 70% and a lack of superior strategies for most AML subtypes (2, 3). New targeted therapy strategies including monoclonal antibodies and small molecule inhibitors are constantly being developed; however to date, none of these targeted therapies have proven more effective than the standard of care with the exception of the use of all-trans retinoic acid (ATRA) in acute promyelocytic leukemia (APL) which has become nearly curable in the majority of cases (4).

Notwithstanding, drug resistance is a major therapeutic challenge in the treatment of AML. Failure of initial therapy can be observed in up to 40% of AML patients, and even when initial therapy is effective, up to 70% of patients eventually succumb to their disease due to aggressive relapse within 5 years (5–7).

**Figure 1:**
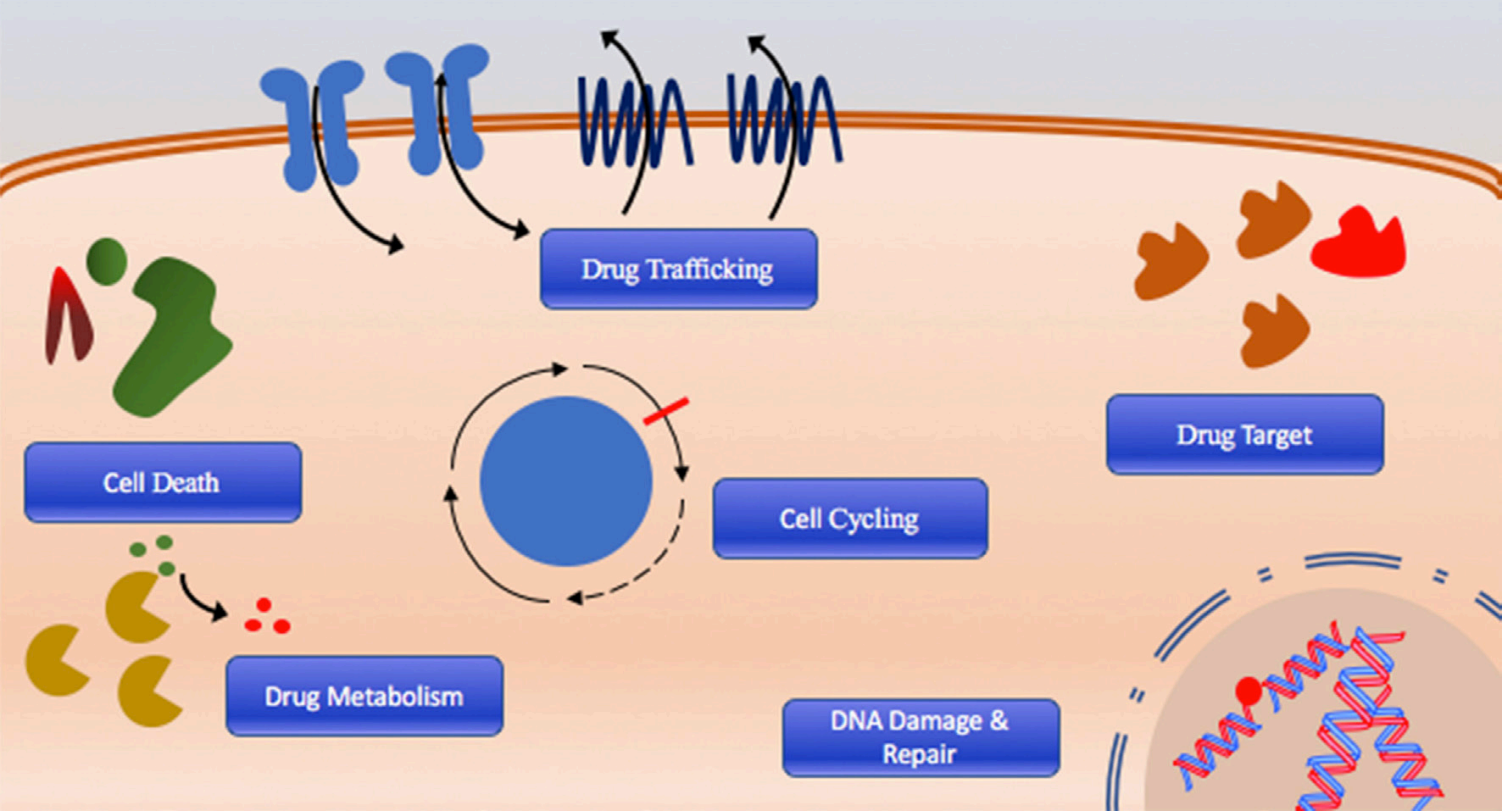
The six hallmarks of drug resistance: DNA damage and repair dysregulation, cell cycle dysregulation, cell death evasion, altered drug metabolism, altered drug target, and dysregulated drug trafficking.

The cause of poor long-term survival is primarily drug resistance, which is either intrinsic in patients that fail initial therapy or acquired after chemotherapy through selection or acquisition of mutations (8). Indeed, relapsed AML is often composed of cells that have distinct molecular and cytogenetic characteristics leading to deficiencies or perturbations in various pathways associated with therapeutic resistance including DNA damage response and drug targeting, cell cycling, cell death, and drug trafficking pathways due to increased or altered drug targets are commonly observed (Figure 1) (8–10). Consequently, outcomes of relapsed disease are abysmal, which highlights a desperate need for novel therapeutic approaches with potential to overcome or prevent therapeutic resistance.

### Non-Coding RNAs (ncRNAs) in AML Therapy Resistance

Among several emerging functions, ncRNAs can act as modulators of gene expression through roles in epigenetics, transcription, translation, as well as homology-dependent post-translational regulation of mRNA transcripts (11). The most widely recognized class of ncRNAs are the microRNAs (miRNAs), which are small 18–24 bp dsRNAs that use cellular RNA-interference machinery to suppress protein expression levels by both degrading or blocking translation of mRNA transcripts (12, 13). It has been convincingly demonstrated in numerous cancers that miRNAs can (1) promote or suppress the development of cancer, (2) be of value in prediction of treatment responses and disease prognosis, and (3) be perturbed as a response to chemotherapy (14, 15).

This review is focused on the small ncRNAs, the miRNAs, in drug resistance; however, long non-coding RNAs (lncRNAs) which are typically >200 bp in length and comprise a large proportion of cellular transcribed RNA have numerous emerging functions in AML pathogenesis (16). lncRNA dysregulation in AML have been reported to have consequences for various cellular processes such as proliferation, survival, and migration (17–19) and have been associated with poor clinical outcome (20–23). Furthermore, lncRNAs signatures associated with well-defined cancer types (24). For instance, *Homeobox (HOX) transcript antisense RNA* (*HOTAIR*) and *HOX antisense intergenic RNA myeloid 1* (*HOTAIRM1*) are substantially upregulated in AML. It was shown in both cell lines and patient samples that the upregulation of *HOTAIR* is specifically associated with indirect upregulation of c-kit through sponging of *miR-193* (20). Recently, doubt has been raised over the prognostic value of *HOTAIR* by Sayad et al.; however, in case–control samples, there was a trend toward clinical significance of *HOTAIR* (25). *HOTAIRM1*, on the other hand, is thought to behave as an endogenous miRNA-sponge for *miR20a, miR-20a/106b*, and *miR-125b* and prevents targeting of *ULK1, E2F1*, and *DRAM2* as demonstrated in luciferase reporter assays (26, 27). In drug resistant AML, however, little is known about the dysregulation of lncRNAs and their respective mechanisms of function.

### miRNA Biology

miRNA derive from the transcription of miRNA loci on genomic DNA by RNA polymerases which create a ~80 nt long transcript primary (pri)-miRNA that are then spliced, capped, polyadenylated, and packaged similar to long-stranded transcripts (28). Further splicing and processing by DROSHA and PASHA transform the pri-miRNA into pre-miRNA. When pre-miRNA exits the nucleus through the function of exportin-5, it is folded into a self-bound hairpin secondary structure known as a “stem-loop” (28, 29). At this stage, the 70–100 nt which make up this stem-loop pre-miRNA is cleaved by a cytoplasmic RNase III such as Dicer into a dsRNA dimer which rapidly breaks down into two strands (29). Depending on the stability of the single strand of miRNA either strand can be active (30–32). A functional third miRNA formed from this complex is thought to originate from the loop region, known as loop-miRNA (33, 34). Next, single-stranded mature miRNAs 19–25 nt in length, bind to the argonaute (Ago) proteins which are one member of a complex of proteins collectively known as the RNA-induced silencing complex (RISC) (35, 36).

Guided by miRNAs, Ago and the RISC move to miRNA recognition elements on mRNA which are commonly, but not limited to non-coding 3′-untranslated regions (3′-UTR) (37, 38). Unlike siRNA, miRNA do not require perfect complementary binding; and only binding to the seed-region appears to be a requirement in most cases (39, 40). This comparatively less stringent binding compared to siRNA allows miRNA to regulate the expression levels of multiple RNA transcripts through target promiscuity (39). Once bound to a target, the endonuclease activity of the RISC is activated *via* the slicer activity of Ago1 (28, 41). Following cleavage, the entire strand is rapidly degraded by endonucleases. Multiple interactions between miRNA and mRNA transcripts are the basis of complex cellular regulatory networks whereby miRNAs control the majority of all protein-coding genes and countless other non-coding genes. In cancer, miRNAs have been demonstrated to play critical roles by modifying or controlling all major hallmarks of cancer including cell division, self-renewal, apoptosis, and DNA damage response among others (42–47).

To date, no comprehensive study examines the role of miRNAs in drug resistant AML. Herein, we describe the miRNAs that have been examined in clinical samples and we highlight miRNA that have been examined mechanistically. Furthermore, we discuss potential miRNA-binding partners of important AML drug resistance machinery found within other cancers to guide future research.

## AML Chemotherapy, DNA Damage, and miRNA Dysregulation

The most common treatment for AML includes an anthracycline like daunorubicin and a nucleoside analog like cytarabine in the “7 + 3” regimen where daunorubicin is administered IV for the first 3 days concomitantly to the IV infusion of cytarabine for 7 days (48, 49). The 7 + 3 regimen is termed *induction therapy* (because of its intent is to induce remission) and has been in place since the 1960s (50). The aim of induction therapy is achieving complete remission (CR), defined clinically as myeloid blast counts in the bone marrow below 5% or minimum residual disease status (49). Efforts to enhance this regimen by escalating dose or adding a third drug has only resulted in increased toxicity with minimal improvement in patient survival. Upon achieving CR, treatment can be consolidated using high doses of cytarabine. Unfortunately, despite undergoing such aggressive chemotherapy regimen with all the associated toxicities and side effect, many patients still relapse within 5 years (48, 49). This is in part due to lack of targeting of leukemic-initiating cells, selection of rare pre-existing resistant AML clones, or the mutagenic effects of the treatments, all of which increase the probability of generating more aggressive AML.

Fundamentally, drug resistance occurs in cells which can evade or withstand treatment. While tumor heterogeneity may explain selection of a pre-existing clone with a favorable mutation, acquired drug resistance is generally defined as the ability of a cell to resist response to the drug to which it was initially responsive. Acquired resistance may be achieved through multiple dosing of the same drug or through as little as a single dose may be explained by the mechanism of drug action.

For instance, anthracyclines used to treat AML such as daunorubicin, doxorubicin, and idarubicin, intercalate DNA, and stall proper DNA replication events (51). Anthracyclines can also target topoisomerase II which normally binds to the scaffold/matrix-associated protein region (S/MAR) to resolve DNA supercoils (52, 53). By binding to topoisomerase II in its open DNA-bound conformation, a stall occurs which can lead to a double-strand break. These double-strand breaks may be fixed aberrantly through non-homologous end joining which can lead to gene mutation. One common mutation in AML, t4:11, occurs at an S/MAR (54–56). This mutation has also been shown to occur in significant proportions in secondary AML patients as well (56). Loss or translocation of the S/MAR may further modulate various miRNAs. As demonstrated by Chavali et al., protein binding to the S/MAR induces histone acetylation that leads to the increased expression of the *miR-17-92* cluster and the miRNAs *miR-221, miR-93, miR-17*, and *let-7b* (57). As DNA damage is most likely to occur in these regions due to daunorubicin, it is likely that dysregulation of miRNA expression can be due to daunorubicin-induced damage directly.

Cytarabine, on the other hand, is a cytosine analog that terminates translation and replication events. It primarily inhibits cells in S phase (DNA replication) but can also inhibit the progression from G_1_ phase into S phase (58, 59). It is known that cytarabine is first metabolized into the triphosphate bound product by deoxycytidine kinase (DCK) and other nucleoside analog enzymes whereby it can then incorporate into the DNA. It is shown that its incorporation can often lead to extensive DNA damage including chromatid breaks (60). Stalled replication forks can also lead to bypass mechanisms such as translesion synthesis (61). This method of DNA replication is more error prone and can lead to mutation events as well. Each of these mechanisms can be demonstrated to have direct or indirect consequences for miRNA function.

**Figure 2:**
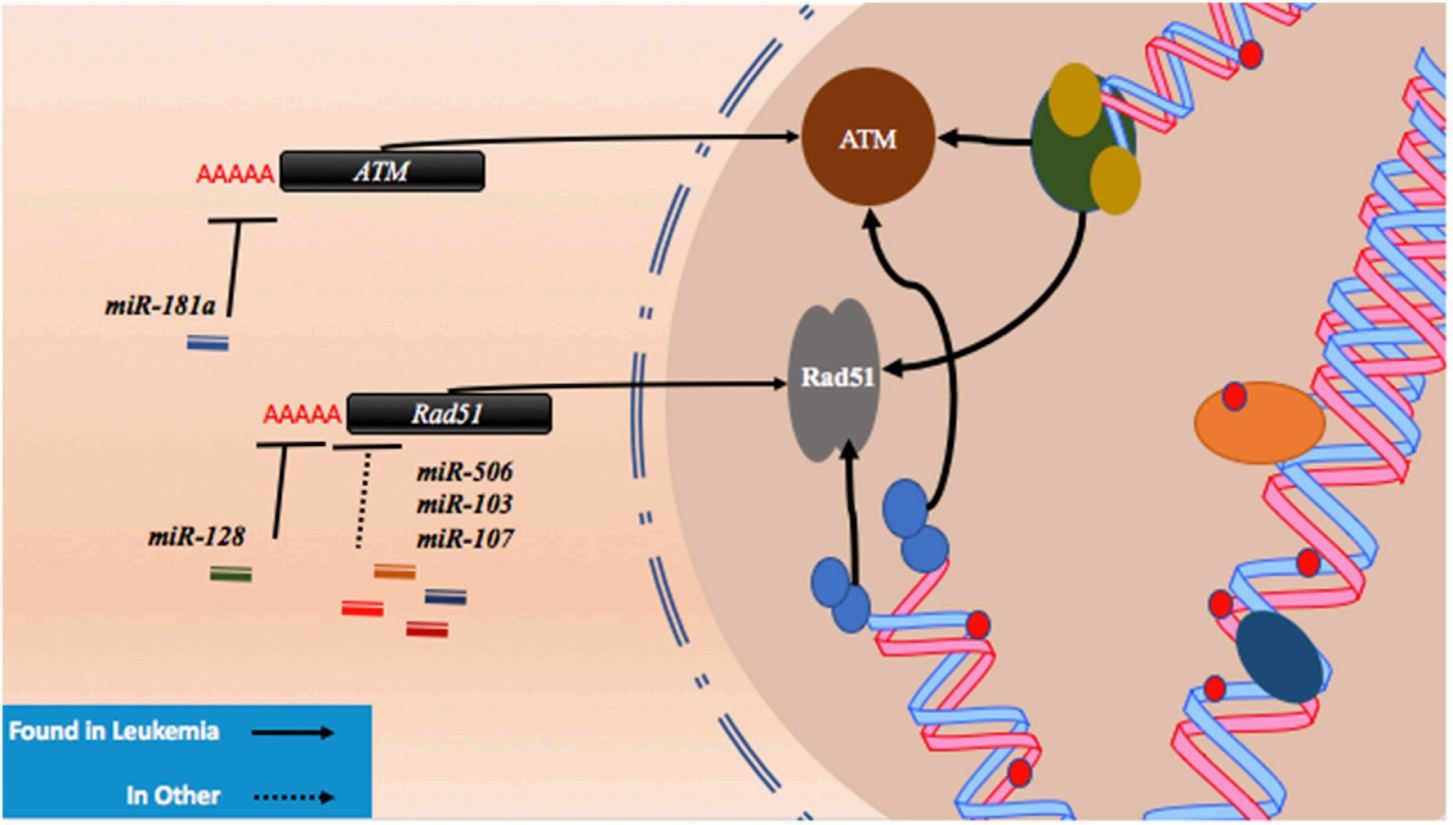
microRNAs (miRNAs) regulate DNA damage response by regulating proteins that behave as DNA damage response elements. In the process of generating DNA damage through genotoxic drugs such as the anthracyclines and the cytosine analogs, the upregulation of effector and response proteins such as ataxia telangiectasia mutated (ATM) and Rad51 is likely to occur. The inhibition of ATM through miR-181a targeting allows tolerance for DNA damage. Reduction of Rad51 through miR-128, miR-506, miR-103, and miR-107 reduces DNA damage response and also contributes to DNA damage tolerance.

As described with both drugs, genotoxic effects can lead to breaks that are then repaired using homologous or non-homologous repair mechanisms leading to miRNA alterations and the upregulation of drug resistance mechanisms. Conversely, miRNA which regulate these associated pathways may also contribute to drug resistance when perturbed by increasing tolerance to DNA damage. For instance, ataxia telangiectasia mutated (ATM) is an important DNA damage sensing and DNA damage response protein that has been demonstrated to contribute to chemoresistance (62). In experiments conducted in leukemic HL60, NB4, and K562 cell lines, it was found that the overexpression of *miR-181a* leads to increased cell proliferation and increased cell cycling through ATM targeting and downregulation (63). Similarly, *miR-128* was reported to affect the propensity for DNA damage in AML cells. In a study conducted in HL60 cells, it was observed that the transfection of *miR-128* led to increased apoptosis, drug sensitivity, and the amount of DNA damage tolerated; however, the mechanism is yet to be elucidated (64). *miR-128* is thought to be upregulated in various cancers, but its levels are reduced in AML cells carrying *NPM1* mutations (Figure 2; Table 1) (65, 66).

**Table 1:** miRNAs demonstrated to directly bind to DNA damage regulatory proteins.

Protein	miRNA	miRNA status in drug resistance	Sample/cancer	Mechanism	Reference
*ATM*	*miR-181a*	Overexpressed	HL60, NB4, K562/AML, CML	ATM downregulation leads to uninhibited growth	Liu et al. (63)
*Rad51*	*miR-128*	Overexpressed	OCI-AML3, MV4-11/AML	Rad51 downregulation leads to increased DNA damage response	Lai et al. (46)
*Rad51*	*miR-506*	Overexpressed	Patient samples/high grade serous ovarian cancer	Rad51 downregulation leads to increased DNA damage response	Liu et al. (47)
*Rad51*	*miR-103*	Overexpressed	U2OS/osteosarcoma	Rad51 downregulation leads to increased DNA damage response	Huang et al. (45)
*Rad51*	*miR-107*	Overexpressed	U2OS/osteosarcoma	Rad51 downregulation leads to increased DNA damage response	Huang et al. (45)

*miRNAs, microRNAs; ATM, ataxia telangiectasia mutated; AML, acute myeloid leukemia*.

Recently, Lai et al. identified a mechanism by which *miR-128* is likely targeting *Rad51* directly and leading to the increased DNA damage response in OCI-AML3 and MV4-11 AML cell lines. In these experiments, *miR-128* led to the sensitization of these cell lines to sapacitabine, a novel oral nucleoside analog prodrug (46). In other cancers, *Rad51* has been shown to be a direct target of other miRNAs such as *miR-506, miR-103*, and *miR-107*. Clinical significance in chemoresistant high-grade serous ovarian cancers was established for *miR-506* while a miRNA mimic library screen revealed *miR-103* and *miR-107* as strong drug resistance contributors in the U2OS cell line, a model for osteosarcoma (Figure 2; Table 1) (45, 47). To date, proteins that are thought to be integral to the activity of anthracyclines and nucleoside analogs such as topoisomerase II and the DNA polymerases are not known to interact with miRNAs. However, topoisomerase II has been demonstrated to be downregulated in drug resistant subtypes of AML (67, 68). miRNA targeting may prove to be a mechanism of topoisomerase II downregulation, but more research is required to establish important links of miRNA-induced dysregulation of DNA repair machinery in drug resistant AML.

## miRNA and Cell Cycling in AML Resistance

The cell cycle represents a series of events that require the input of various checkpoint proteins known as cyclins and cyclin-dependent kinases (CDK) to proceed into division (69). These proteins, in turn, receive input from DNA damage sensing proteins such as ATM/ATR and CHK1/2 (70, 71). The majority of rapidly dividing cancer cells can be found in one of two major phases: the interphase; which consists of G1, S phase (DNA replication) followed by G2; and the M phase, where cells undergo mitosis. Cell cycle manipulation can be a drug resistance mechanism as cell cycle arrest at different phases or quiescence can lead to chemotherapy evasion; however, increased proliferation can also contribute to resistance (72–75).

The process of cell division begins in G1 by the duplication of various proteins, chromatin remodeling, and the verification that the DNA is free of DNA damage. In a healthy cell, if substantial levels of DNA damage are found, ATM/ATR become activated leading to eventual CDK2 inhibition and arrest at the G1/S checkpoint through p21 signaling, where the mechanisms of action of many miRNAs have been elucidated (76). *CDK2* has been found to be inhibited by *miR-638*, where it was demonstrated in HL-60, NB4, and THP-1 that an upregulation of *miR-638* leads to a reduction in cell cycling and a differentiation block in APL (77). The differentiation block was found to occur at the G1/S checkpoint and differentiation inducers like ATRA were found to be more effective in cells with *miR-638* downregulation (77).

**Figure 3:**
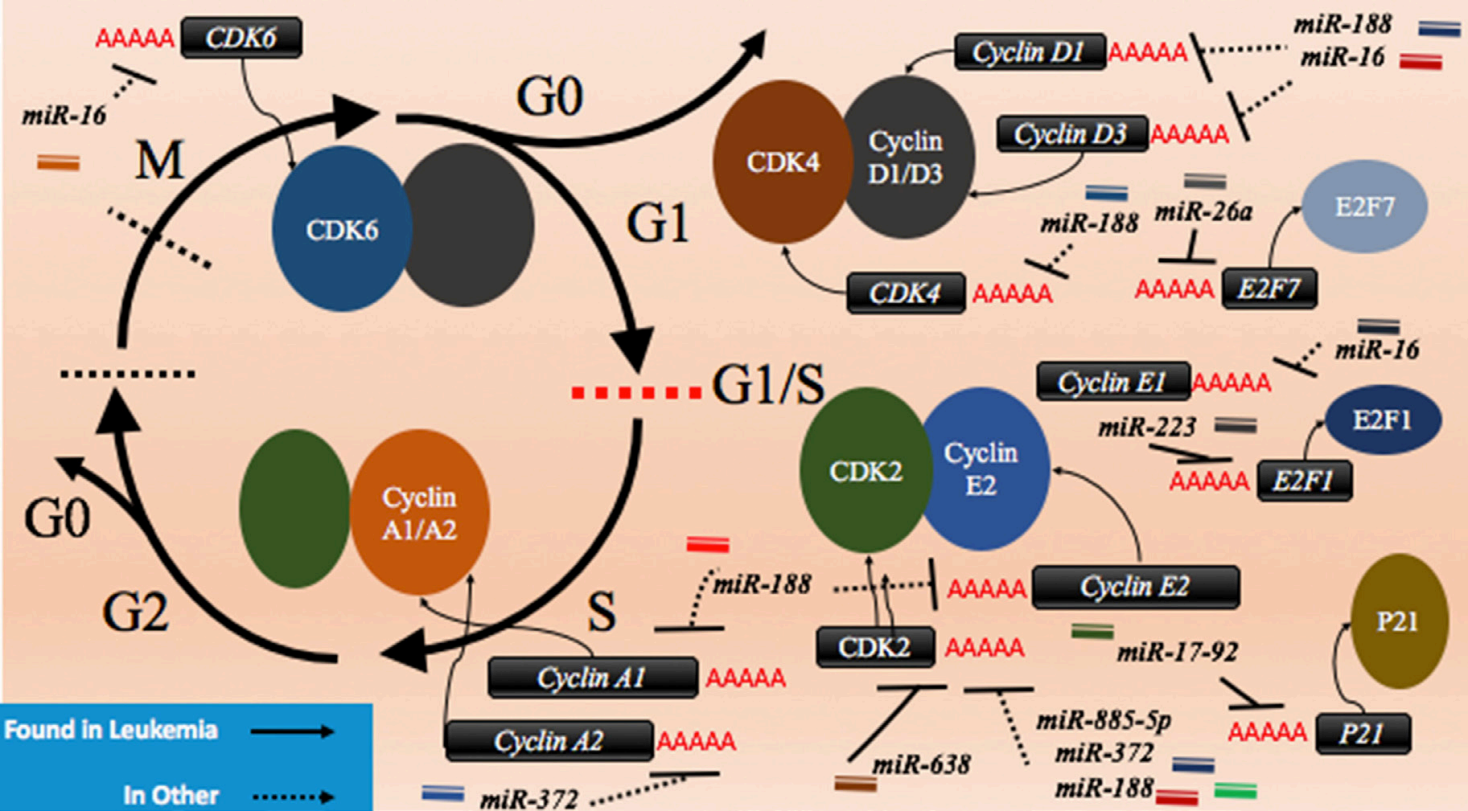
microRNAs (miRNAs) can dysregulate cell cycling mechanisms by dysregulating several phases of the cell cycle, but the majority of known targeting occurs at the G1 and S phases and at the G1/S transition. The downregulation of the cyclins that would normally signal for cell cycling to proceed can be downregulated. Cyclin D1 and cyclin D3 can be dysregulated by miR-188 and miR-16, cyclin E1 can be knocked down by miR-16 while cyclin E2 can be downregulated by miR-17-92 and finally, cyclin A1 and A2 are downregulated by miR-188 and miR-372, respectively. The cyclin-dependent kinases (CDKs) are also adjustable through miRNA targeting and their targeting reduces cycling as well. CDK2 can be downregulated by miR-638, miR-885-5p, miR-372, and miR-188; CDK4 is downregulated by miR-188, and CDK6 is downregulated by miR-16. Effector proteins such as E2F1, E2F7, and p21 can also be downregulated by miRNAs to lead to differentiation blocks. They can be targeted by miR-223, miR-26a, and miR-17-92, respectively.

*CDK2* has been demonstrated to be a target of various miRNAs in cancer including *miR-885-5p, miR-372*, and *miR-188* (Figure 3; Table 2). In contrast to *miR-638* in AML, *miR-885-5p* was demonstrated to play a tumor suppressive role in neuroblastoma by inhibiting *CDK2* and promoting senescence and apoptosis (78). *miR-372* demonstrated targeting of both *CDK2* and cyclin A1, which is highly expressed during S phase. Like *miR-885-5p, miR-372* was demonstrated to play a tumor suppressive role as demonstrated in HeLa cells and tissue samples of cervical cancer (79). *miR-188* was demonstrated to directly bind several genes which play a role in cycling such as cyclin D1, cyclin D3, cyclin A2, cyclin E1, CDK2, and CDK4 with varying degrees and it demonstrated modest knockdown of CDK2 relative to the other genes (80). In this study, it was found that the arrest occurs at the G1/S transition and that *miR-188* plays a tumor suppressive role (80).

**Table 2:** Cell cycling gene dysregulations induced by miRNA binding.

Protein	miRNA	miRNA status in drug resistance	Sample/cancer	Mechanism	Reference
*CDK2*	*miR-638*	Overexpressed	HL60, NB4, THP-1/APL	CDK2 downregulation prevents G1/S progression	Lin et al. (77)
*CDK2*	*miR-885-5p*	Reduction	Patient samples, SH-EP, KELLY, IMR32, SK-N-BE(2)c, and HDN 33 cell lines/neuroblastoma	Reduced cycling promotes senescence	Afanasyeva et al. (78)
*CDK2*	*miR-372*	Reduced	HeLa/cervical cancer	Reduced cycling prevents cell growth	Tian et al. (79)
*CDK2*	*miR-188*	Reduced	CNE cells/nasopharyngeal carcinoma	G1/S arrest prevents cell cycling	Wu et al. (80)
*Cyclin A1*	*miR-372*	Reduced	HeLa/cervical cancer	Reduced cycling prevents cell growth	Tian et al. (79)
*Cyclin D1*	*miR-188*	Reduced	CNE cells/nasopharyngeal carcinoma	G1/S arrest prevents cell cycling	Wu et al. (80)
*Cyclin D1*	*miR-16*	Reduced	A549/lung cancer	G1 and G1/S arrest reduces proliferation	Liu et al. (81)
*Cyclin D3*	*miR-188*	Reduced	CNE cells/nasopharyngeal carcinoma	G1/S arrest prevents cell cycling	Wu et al. (80)
*Cyclin D3*	*miR-16*	Reduced	A549/lung cancer	G1 and G1/S arrest reduces proliferation	Liu et al. (81)
*Cyclin A2*	*miR-188*	Reduced	CNE cells/nasopharyngeal carcinoma	G1/S arrest prevents cell cycling	Wu et al. (80)
*Cyclin E2*	*miR-188*	Reduced	CNE cells/nasopharyngeal carcinoma	G1/S arrest prevents cell cycling	Wu et al. (80)
*Cdk4*	*miR-188*	Reduced	CNE cells/nasopharyngeal carcinoma	G1/S arrest prevents cell cycling	Wu et al. (80)
*Cdk6*	*miR-16*	Reduced	A549/lung cancer	G1 and G1/S arrest reduces proliferation	Liu et al. (81)
*Cyclin E1*	*miR-16*	Reduced	A549/lung cancer	miR-16 loss may lead to G1 and G1/S arrest reduces proliferation	Liu et al. (81)
*E2F7*	*miR-26a*	Reduced	Patient samples, HL60, U937/APL	Downregulation of E2F7 reduces progression	Salvatori et al. (82)
*P21*	*miR-17-92*	Overexpressed	MLL transformed cells/AML	Downregulation of p21 promotes non-differentiation	Wong et al. (83)
*E2F1*	*miR-223*	Overexpressed	Patient samples, K562, U937/AML, CML	E2F1 downregulation contributes to non-differentiated cell cycle progression	Pulikkan et al. (84)

*miRNAs, microRNAs; APL, acute promyelocytic leukemia; CDK, cyclin-dependent kinase; AML, acute myeloid leukemia*.

**Figure 4:**
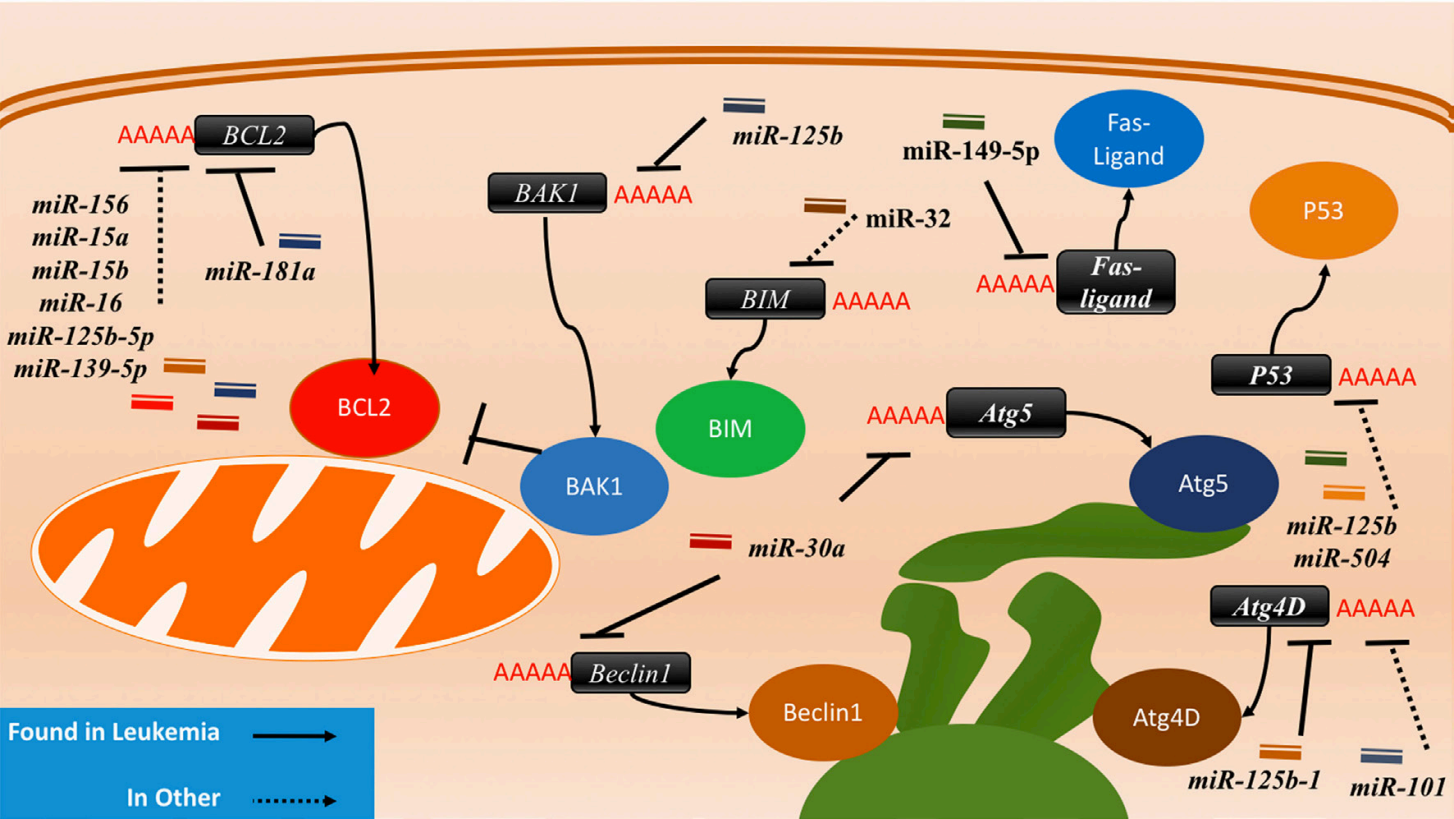
The interactions between microRNAs (miRNAs) and cell death-related proteins in drug resistant cells. Within the apoptosis cell death mechanism, proteins part of the intrinsic or extrinsic pathway can respond to miRNAs to inhibit apoptosis or reduce their regulatory signaling of apoptosis. BCL2, an anti-apoptosis gene, will gain signaling when the associated miRNAs such as miR-156, miR-15a/b, miR-16, miR-125b-5p, and miR-139-5p are lost in the drug resistant cell. The gain of BAK1 miRNA targeting through miR-125b or the gain of BIM targeting through miR-32 will lead to the same effect as well. The Fas-ligand can also be suppressed by miR-149-5p thus ending extrinsic apoptosis signaling. P53 suppression through miR-125b and miR-504 will prevent apoptosis as well. Dysregulating autophagy through increased targeting may increase drug resistance through the binding of miR-125b and miR-101 on Atg4D. miR-30a is known to inversely correlate with Beclin1 and Atg5 in leukemia cell lines, but less is known about the outcome of this interaction.

Other miRNAs such as the *miR-16* family members famously known for downregulation of *BCL2* (Figure 4) are also shown to simultaneously directly target several cycling genes such as *cyclin D1, cyclin D3, cyclin E1*, and *CDK6* (Figure 3; Table 2). As demonstrated in the A549 cell line by Liu et al., this targeting and likely the targeting of downstream effectors leads to the arrest in G1 and at G1/S, a phenomenon observed by others (81, 85, 86). The targeting of *Cyclin E* has since been demonstrated as playing an important role in certain cancers such as cervical cancer and breast cancer (86–89). The *miR-15* and *miR-16* family may be response elements of E2F1 and as such, may be contributing to a feedback mechanism (90).

The transcription factor *E2F* family may also be a target of miRNAs. *E2F7*, a transcriptional response element gene implicated in cell cycling, is downregulated by *miR-26a* in AML (82). This inhibition in turn reduces *c-myc* transcriptional activation and sequential *miR-17-92* reduced transcription, which has previously been implicated in promoting a differentiation block (82, 91, 92). When active, *miR-17-92* members may be in part directly targeting p21 and promoting cycling, as demonstrated in MLL transformed leukemic cells by Wong et al. (83). The inhibition of *E2F7* may lead to a reduction of miRNAs involved in proliferation such as *miR-25, miR*-*26a, miR-27b, miR-92a*, and *miR-7* thus behaving as a regulatory mechanism (93).

In other instances, miRNAs can behave as direct inhibitors of their own transcriptional repressor thus behaving as autoregulatory elements. It has been demonstrated by Pulikkan et al. that this is the case for *miR-223* and *E2F1* regulation (84). E2F1, an important response element in G1/S, can repress transcription of *miR-223* which in turn can repress *E2F1* (84, 94, 95). The differentiation block observed in APL may be further exacerbated by miRNAs like *miR-223* (Figure 3; Table 2). The complexity of interactions within miRNA–mRNA networks demonstrates the need for further analyses elucidating the major pathways of feedback and feedforward signaling.

## Cell Death and miRNA

In the majority of blast cells that experience sufficient levels of DNA damage upon chemotherapy, programmed cell death (PCD) will become activated. PCD may take the form of apoptosis or autophagy. Apoptosis is characterized by specific changes in morphology such as cell shrinkage and pyknosis (96). Autophagy, on the other hand, is characterized by cellular degradation and the re-introduction of catabolic products into anabolic processes (97, 98). Autophagy can play both a detrimental and a beneficial role in cancer cells and it can also contribute to the generation of leukemia (98–100). Apoptosis, on the other hand, while it is an essential component of normal cell turnover, only its downregulation will often be a major contributor for aberrant cancer growth and its further suppression can lead to drug resistance.

### miRNA and BCL2 Family Members

miRNA-associated dysregulation of apoptosis has been observed in drug resistant AML cells. Given that AML is often characterized by aberrant DNA repair and maintenance, tolerance of these damaged lesions is observed through the downregulation of pro-apoptotic markers and damage sensors, or the upregulation of antiapoptotic factors. Of the apoptosis-related families, the BCL2 protein family is the most well described in miRNA dysregulation driven in AML. The BCL2 protein itself is commonly considered as a crucial anti-apoptosis gene as it inhibits the mitochondrial pro-apoptotic proteins such as Bak and Bax. While it can be dysregulated or mutated in cancers, it is observed that dysregulation may also occur in the development of drug resistance. Many miRNAs including *miR-15/miR-16, miR-125b-5p, miR-139-5p, miR-145*, and *miR-181a* have been shown to suppress the translation of *BCL2* and decrease the propensity for activation of apoptosis (Figure 4; Table 3).

**Table 3:** The interactions of miRNAs with cell death-related proteins.

Protein	miRNA	miRNA status in drug resistance	Sample/cancer	Mechanism	Reference
*BCL2*	*miR-181a*	Reduced	K562/CML HL60/APL	Reduced miR-181a leads to increased apoptosis suppression Cytarabine resistance presents with reduced miR-181a expression and apoptosis suppression	Li et al. (101) Bai et al. (102)

*BCL2*	*miR-15b*	Reduced	SG7901 cells/gastric cancer	Reduced miR-15b expression leads to BCL2 overexpression and apoptosis suppression	Xia et al. (103)

*BCL2*	*miR-16*	Reduced	SG7901 cells/gastric cancer ERΔ16 MCF7/breast cancer U251MG, AM38	Reduced miR-16 expression leads to BCL2 overexpression and apoptosis suppression	Xia et al. (103) Cittelly et al. (104) Han and Chen (105)

*BCL2*	*miR-15a*	Reduced	HERΔ16 MCF7/breast cancer	Downregulated miR-15a leads to BCL2 overexpression	Cittelly et al. (104)

*BCL2*	*miR-125b-5p*	Reduced	Patient samples/gallbladder cancer	Downregulation of miR-125b-5p disinhibits BCL2 and leads to anti-apoptosis	Yang et al. (106)

*BCL2*	*miR-139-5p*	Reduced	Colorectal cancer	Downregulation of miR-139-5p leads to BCL2 disinhibition and anti-apoptosis	Li et al. (107)

*BAK1*	*miR-125b*	Overexpressed	HL60, NB4/APL NB4, K562/CML MDA-MB-435, MDA-MB-231/breast cancer HMLE/breast cancer PC-3466C, LNCaP/prostate cancer	Suppression of Bak1 leads to apoptosis avoidance	Zhang et al. (7) Li et al. (108) Zhou et al. (109) Shi et al. (110)

*BIM*	*miR-32*	Overexpressed	LNCaP/prostate cancer	Downregulation of *BIM* leads to apoptosis evasion	Gocek et al. (111)

*p53*	*miR-125b*	Overexpressed	SH-SY5Y/neuroblastoma	Direct binding to the *P53* by *miR-125b* leads to further inhibition of apoptosis response	Le et al. (112)

*p53*	*miR-504*	Overexpressed	HCT116 (colorectal carcinoma), H460 (large cell lung cancer), MCF-7 (ER + breast cancer), U2OS (osteosarcoma), A498 (kidney carcinoma)	Direct binding by *miR-504* reduces the propensity of a cell to enter apoptosis	Hu et al. (113)

*Fas-ligand*	*miR-149-5p*	Overexpressed	THP-1/AML	Downregulation of the *Fas-ligand* reduces activation of the extrinsic apoptosis pathway	Tian and Yan (114)

*Beclin 1*	*miR-30a*	Unknown	K562/CML	Inverse correlation found	Yu et al. (115)

*ATG5*	*miR-30a*	Unknown	K562/CML	Inverse correlation found	Yu et al. (115)

*ATG4D*	*miR-125b1*	Overexpressed	NB4/APL	Inhibition of autophagy	Zeng et al. (116)

*ATG4D*	*miR-101*	Overexpressed	MCF7/breast cancer	Inhibition of autophagy contributed to tamoxifen resistance	Frankel et al. (117)

*miRNAs, microRNAs; APL, acute promyelocytic leukemial; AML, acute myeloid leukemia*.

Of the *BCL2*-targeting miRNAs, only *miR-181a* has been shown to do so in AML cells. In K562 CML cells, it was demonstrated by Li et al. that the drug resistant form had 40% of the *miR-181a* levels found in the parental cell line. When the parental cells were transfected with a *miR-181a* inhibitor, resistance developed (101). In a separate study conducted by Bai et al. in cytarabine resistant HL60, it was found that the resistance phenotype can be also be attributed to reduced *BCL2* targeting by *miR-181a*, whereas its ectopic expression sensitizes the cells to treatment to cytarabine (102). Other studies of *miR-181a* in AML have also demonstrated that it is often downregulated in drug resistant AML, that it can serve as an independent prognostic marker and potentially modulate the interaction with natural killer cells as well (118–122). In molecular poor risk group AML with *FLT3-ITD* mutations, it was demonstrated that high *miR-181a* also strongly predicted better survival (123).

The *miR-15/16* have been shown to suppress BCL2 in multiple cancers including gastric cancer, breast cancer, and glioma and the loss of this locus has also been observed in CLL (124–127). Xia et al. demonstrated that *miR-15b* and *miR-16* are lost in vincristine resistant SGC7901 cells, a gastric cell line (103). Cittelly et al. later demonstrated that in a common mutation of the *HER2* gene, *HER*Δ*16*, representative of 30% of HER2 dysregulations in estrogen receptor positive breast cancers, the downregulation of *miR-15a* and *miR-16* is observed (104). In MCF-7 cells ectopically expressing this mutant variant, it was shown that tamoxifen resistance may be in part due to the reduced regulation of *BCL2* by *miR-15a* and *miR-16*, which leads to apoptosis evasion (104). In glioma cells that are resistant to temozolomide, it was demonstrated that the loss of *miR-16* specifically can contribute to resistance in the U251MG/Temozolomide resistant cell line and that the blocking of *miR-16* in the temozolomide sensitive AM38 cell line increased resistance by de-repressing *BCL2* (105).

In a genome-wide gene expression analysis of gallbladder cancer clinical samples, *miR-125b-5p* was found to be statistically downregulated in cisplatin resistant patients (*N* = 6). Analyses demonstrated that this miRNA can directly bind to the 3′UTR of *BCL2*, contribute to cisplatin desensitization, and increase tumor formation in mice (106). A similar analysis of patient samples conducted in colorectal cancer demonstrated that *miR-139-5p* inhibits the epithelial-to-mesenchymal transition and contributes to drug resistance by downregulating *BCL2* (107). Bioinformatic studies also demonstrate binding of other miRNAs to the *BCL2* mRNA as putative mechanisms of miRNA-induced downregulations. For instance, bioinformatic analysis of *miR-451* through miRBase and miRanda identified it as an inhibitor of *BCL2* (128). Similarly, in paclitaxel-resistant breast cancer, it was demonstrated that *miR-451* may also inhibit BCL2.

The BCL2 antagonist/killer 1 (Bak1) protein is upregulated in the progression of apoptosis in normal cells; in drug resistant cancers, however, it is observed that there is *Bak1* suppression through *miR-125b* binding. The binding of *miR-125b* to the *Bak1* transcript was initially examined in the prostate cancer cell lines PC-346C and LNCaP in the context of androgen-independent signaling, but effect on drug resistance was not examined (110). In APL, *miR-125b* was demonstrated to be clinically relevant, in CML mice models, and it was further demonstrated that direct suppression occurs in the cell lines NB4, HL60, and K562 (7, 108). A similar link between *miR-125b* and *Bak1* was established in MDA-MB-435 and MDA-MB-231 where it was demonstrated that *miR-125b* is capable of *Bak1* suppression in Taxol resistant cells (109). The mechanism of *miR-125b* upregulation was further elucidated to be through Wnt signaling and specifically through Snail binding; an upregulation thought to also occur in cancer stem cells (129).

The Bcl-2-like protein 11, also known as, BIM, has been demonstrated to be a direct target of *miR-32* in a previous study in LNCaP prostate cancer cells. This pro-apoptotic protein can be downregulated by *miR-32* and consequently lead to resistance and increased cell proliferation (130). Studies in the AML cell lines HL60 and U937 also demonstrated an inverse correlation between *miR-32* and *BIM* (111).

### miRNA and P53 Regulation

The tumor-suppressor protein p53, often referred to as guardian of the genome is dysregulated in 50% of all cancers. In *wild-type* cells, p53 is often suppressed and destabilized by mdm2, mdm4, and mdmx which behave like E3 ligases, marking P53 by ubiquitination for degradation. Phosphorylation of p53 by ATM leads to its stabilization and release from the mdm protein family. p53 can then behave as a transcription factor by activating apoptosis-related genes (both intrinsic and extrinsic), cell cycle arrest related genes or DNA repair related genes and it can directly bind to the mitochondria to participate in membrane permeabilization (131, 132).

*P53* has been identified as a direct target of miRNA binding by *miR-125b* and *miR-504. miR-125b* was shown to directly decrease P53 transcript levels and consequently decrease apoptosis response to irradiation in neuroblastoma cells and in lung fibroblasts (Figure 4; Table 3) (112). *miR-504* was first computationally predicted and then demonstrated in various cell lines including HCT116 (colorectal carcinoma), H460 (large cell lung cancer), MCF-7 (ER + breast cancer), U2OS (osteosarcoma), and A498 (kidney carcinoma) cells to directly target the 3′UTR of *P53* (113). *P53* is also importantly downregulated through indirect ways by *miR-34a*, which is thought to play a crucial role in P53’s pro-apoptotic abilities (133, 134). It has been demonstrated that *miR-34a* can indirectly increase P53 by inhibiting P53 negative regulators such as *SIRT1* in colon cancer as demonstrated by Yamakuchi et al. and likely through binding of *mdm4* as well, as predicted bioinformatically (135–137).

Furthermore, it has been demonstrated that P53 transcriptionally activates *miR-34a* which in turn modulates and fine tunes P53’s signal (134). Consequently, the relationship between *miR-34a* and P53 is context dependent as the mutation status of *P53* can influence the response and outcome of *miR-34a* activity (138). In the study conducted by Rücker et al., it was found that P53 alterations were the most common molecular lesions which coincided with complex karyotypes in AML (138). Low *miR-34a* and P53 alterations were shown to have the poorest clinical outcome in terms of drug resistance and survival. The low expression was shown to also correlate with a specific gene expression profile consisting of P53-associated proteins. In complex karyotypes that did not have a *P53* alteration, high *miR-34a* predicted a poor overall survival while loss of *P53* and high *miR-34a* predicted better outcome (138). The interplay between *miR-34a* and *P53* demonstrates that the same miRNA can have opposite effects depending on the mutation status of the associated mRNA and highlights the necessity of describing miRNA activity in relation to the activity of associated mRNA.

### Other Apoptosis-Related Proteins

For the apoptotic extrinsic pathway, it was reported by Tian et al. that *miR-149-5p* can directly downregulate the *Fas-ligand* and reduce the levels of the apoptosis effector proteins caspase-8, caspase-2, and caspase-3; however, no effect on drug resistance is demonstrated (114). It is possible that *miR-181a* and *miR-21* can suppress the Fas-ligand in cancers as they are shown to interact with the Fas-ligand in bone marrow-derived mesenchymal cells and cardiomyocytes, respectively (139, 140). The binding of miRNAs to caspases has also not been examined closely in cancers, but in an experiment conducted by Zhang et al. in endothelial cells demonstrated *caspase-3* downregulation due to *let-7g* inhibition. As such, this targeting reduced the progression of apoptosis and lead to higher tolerance of oxidative stress (141).

### Autophagy and miRNA

Autophagy is regulated by many autophagy related (ATG) proteins which play various roles in the formation of the autophagosome (100, 142). It has been observed that miRNAs can likely play a role in autophagy and that AML cells can have dysregulated autophagy (97). To date, two miRNAs have been found to associate with autophagy in leukemia: *miR-30a* and *miR-125b1* (115, 116). *miR-30a* is inversely correlated with *Beclin1* and *ATG5* in K562, but direct binding and relevance to drug resistance is yet to be demonstrated (115). *miR-125b1*, on the other hand, can bind *RAM2, ATG4D*, and *UVRAG* as demonstrated in NB4 cells (116). The activity of *miR-125b1* in this circumstance contributed to inhibition of autophagy through *ATG4D*. In other cancers, *ATG4D* was found to be a direct target of *miR-101* and its inhibition may contribute to 4-hydroxytamoxifen sensitization in the breast cancer lines MCF7 and T47D (117).

## Drug Metabolism and Chemoresistance

Drug activation and drug clearance can be altered in cells to reduce the effective dose of the drug. These proteins are highly varied, but can largely be characterized into two major classes: the phase I and the phase II class of enzymes. Phase I enzymes typically perform redox reactions or hydrolysis reactions. While they often precede phase II enzyme activity, this is not always required. Phase II enzymes typically increase the polarity of the molecule through the addition of a sub-group such as UDP-glucoronate, sulfate, methane, acetate, or glutathione (143).

Anthracyclines are active drugs that can carry out their genotoxic effects directly. Their metabolism into the semiquinone form, the hydroxyaglycone form, deoxyaglycone form, or the alcohol form will decrease its likelihood of intercalating DNA as it reduces the anthracycline’s lipophilicity. It is unclear whether the anthracyclines lose efficacy through metabolism. As demonstrated from cardiotoxicity assays in rat and rabbit, the metabolites may have differing effects depending on the organism in question and the rate of metabolism. In rats, the alcohol form may retain some activity, but the effects of the active drug are more pronounced (143, 144). In rabbits, the alcohol derivative is implicated in the cardiotoxic effects of the anthracyclines (143, 145). It is thought that the enzymes CBR1/3 and AKR1A1/C3 can act on the parent drug to form the alcohol form. The hydroxaglycone and the deoxyaglycone forms can be generated in part by certain cytochrome P450 (CYP) enzymes such as CYP3A4/5, CYP2D6, xanthine dehydrogenase (XDH), and NAD(P)H quinone dehydrogenase 1 (NQO1) (146–150). XDH, NQO1 along with nitric oxide synthase can help in generating the semiquinone form (151–153).

**Figure 5:**
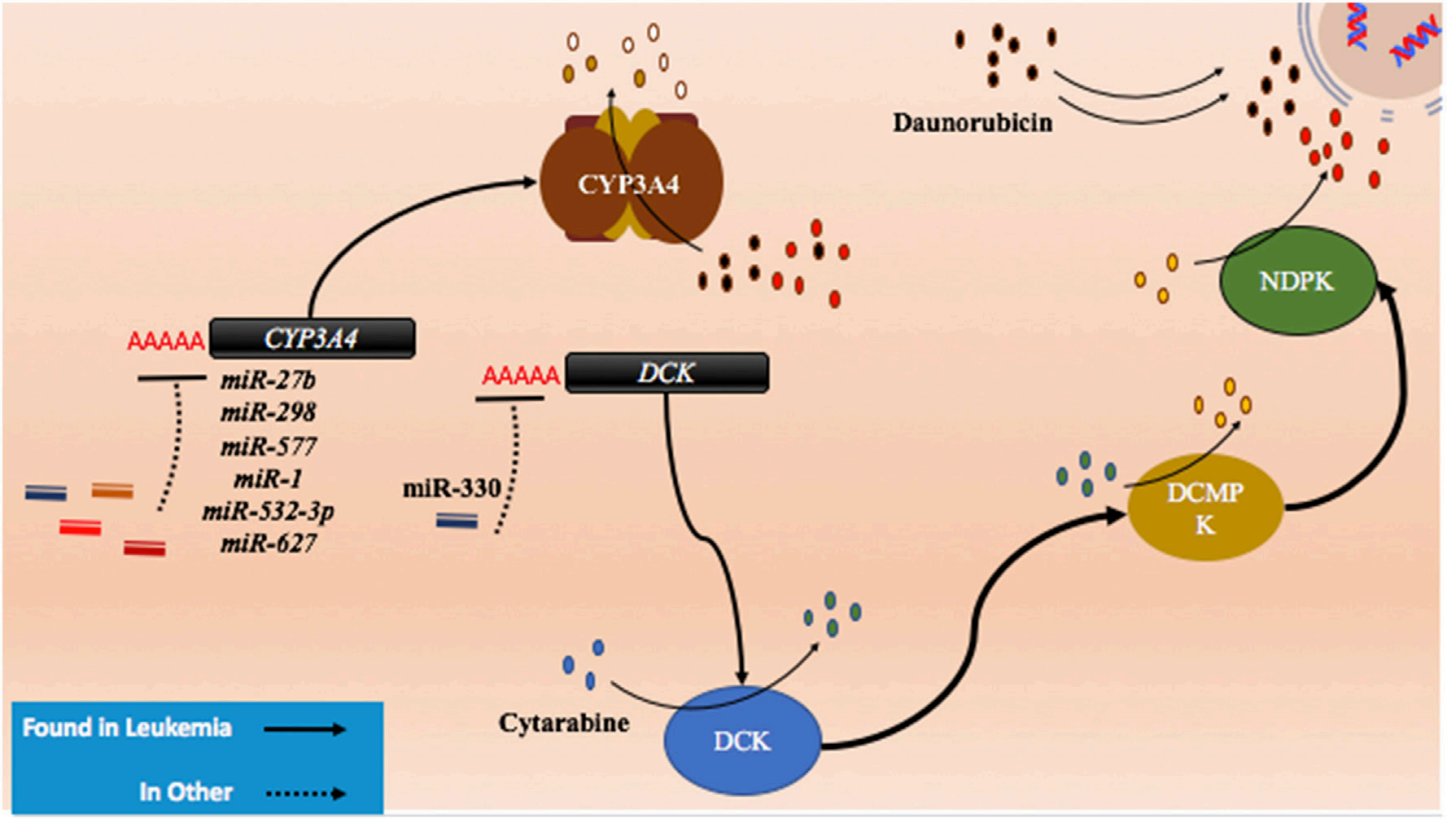
The role of metabolism and microRNA (miRNA) in daunorubicin and cytarabine treatment. While daunorubicin is an active drug, cytosine requires bio-activation. As a cytosine analog, it must undergo three phosphorylation steps to become fully activated and capable of incorporating into the genome. The deactivation of daunorubicin and cytarabine is partially dependent on the cytochrome P450s and they commonly share CYP3A4 in their pathway of degradation. In other cancer, CYP3A4 has been shown to be targeted by miR-27b, miR-298, miR-577, miR-1, miR-532-3p, and miR-627. In the pathway of cytarabine activation, deoxycytidine kinase (DCK) has been shown to be downregulated by miR-330 in other cancers.

Cytarabine and other nucleoside analogs require phosphorylation through DNA/RNA synthesizing enzymes such as the nucleoside kinases to become candidates for incorporation into nascent DNA. Cytarabine requires activation by several enzymes including deoxycytidine monophosphate kinase, nucleoside diphosphate kinase, and the rate limiting DCK (Figure 5) (154, 155). It is then metabolized by various enzymes including CYP3A4, 5′ nucleotidase, cytidine deaminase, and deoxycytidylate deaminase (154, 155).

Currently, there are few publications that highlight the role of miRNAs in anthracycline and cytosine analog metabolizing enzymes in AML. However, certain miRNAs such as *miR-27b* and *miR-298* have demonstrated direct binding of *CYP3A4* in a pancreatic cell line and *miR-577, miR-1, miR-532-3p*, and *miR-627* were found to target *CYP3A4* in HEK 293T cells (Figure 5; Table 4) (156, 157). In gemcitabine resistant colon and lung cancer cells, Hodzic et al. established a correlation between *miRNA-330* and *DCK* expression levels (158). Further studies interrogating the role of computationally predicted miRNAs and miRNAs discovered in other cancer subtypes may help establish a role for miRNAs in metabolism in drug resistant AML.

**Table 4:** miRNA targeting proteins involved in drug metabolism.

Protein	miRNA	miRNA status in drug resistance	Sample/cancer	Mechanism	Reference
*CYP3A4*	*miR-27b*	Overexpressed	LS-180, PANC 1/colon adenocarcinoma and pancreatic cancer	Inhibition of CYP3A4 lead to reduced activation of cyclophosphamide and reduced sensitivity	Pan et al. (156)
*CYP3A4*	*miR-298*	Overexpressed	LS-180, PANC 1/colon adenocarcinoma and pancreatic cancer	Inhibition of CYP3A4 lead to reduced activation of cyclophosphamide and reduced sensitivity	Wei et al. (157)
*CYP3A4*	*miR-577*	Overexpressed	HEK 293T/cancer	Inhibition of CYP3A4 lead to reduced activation of cyclophosphamide and reduced sensitivity	Wei et al. (157)
*CYP3A4*	*miR-1*	Overexpressed	HEK 293T/cancer	Inhibition of CYP3A4 lead to reduced activation of cyclophosphamide and reduced sensitivity	Wei et al. (157)
*CYP3A4*	*miR-532-3p*	Overexpressed	HEK 293T/cancer	Inhibition of CYP3A4 lead to reduced activation of cyclophosphamide and reduced sensitivity	Wei et al. (157)
*CYP3A4*	*miR-627*	Overexpressed	HEK 293T/cancer	Inhibition of CYP3A4 lead to reduced activation of cyclophosphamide and reduced sensitivity	Wei et al. (157)
*DCK*	*miR-330*	Overexpressed	HEK 293T/cancer	Inverse correlation between miRNA-mRNA suggests interaction	Hodzic et al. (158)

*miRNAs, microRNAs; DCK, deoxycytidine kinase*.

## Drug Trafficking and miRNA in Chemoresistance

The trafficking of the anticancer drugs can dramatically modulate treatment response as a reduction in influx or an increase in efflux will reduce the effective intracellular concentration of drug. Due to the lipophilicity of the anthracyclines, they can freely diffuse into the cell, but they can also bind to the SLC22A16 solute pump to enter cells (153, 159–161). While there are some reports that suggest the role of SLC22A16 in bleomycin resistance, the role of this transporter in anthracycline resistance is yet to be explored (162, 163). As such, while there are predicted miRNA-binding sites on this protein, none are yet confirmed.

Cytarabine and other cytosine analogs, on the other hand, necessitate the function of nucleoside transporters to enter the cell. The nucleoside transporters are composed of six major protein families: human equilibrative nucleoside transporters (hENTs) and human concentrative nucleoside transporters (hCNTs), organic anion transporters, organic cation transporters, peptide transporters, and the multidrug resistance protein family (MRP), with the hCNTs and hENTs playing the most major role of cytarabine import (164–166). In childhood leukemia, the hENT protein family has demonstrated to correlate with cytarabine resistance, but miRNA-mediated mechanisms are yet to be confirmed (167, 168).

**Figure 6:**
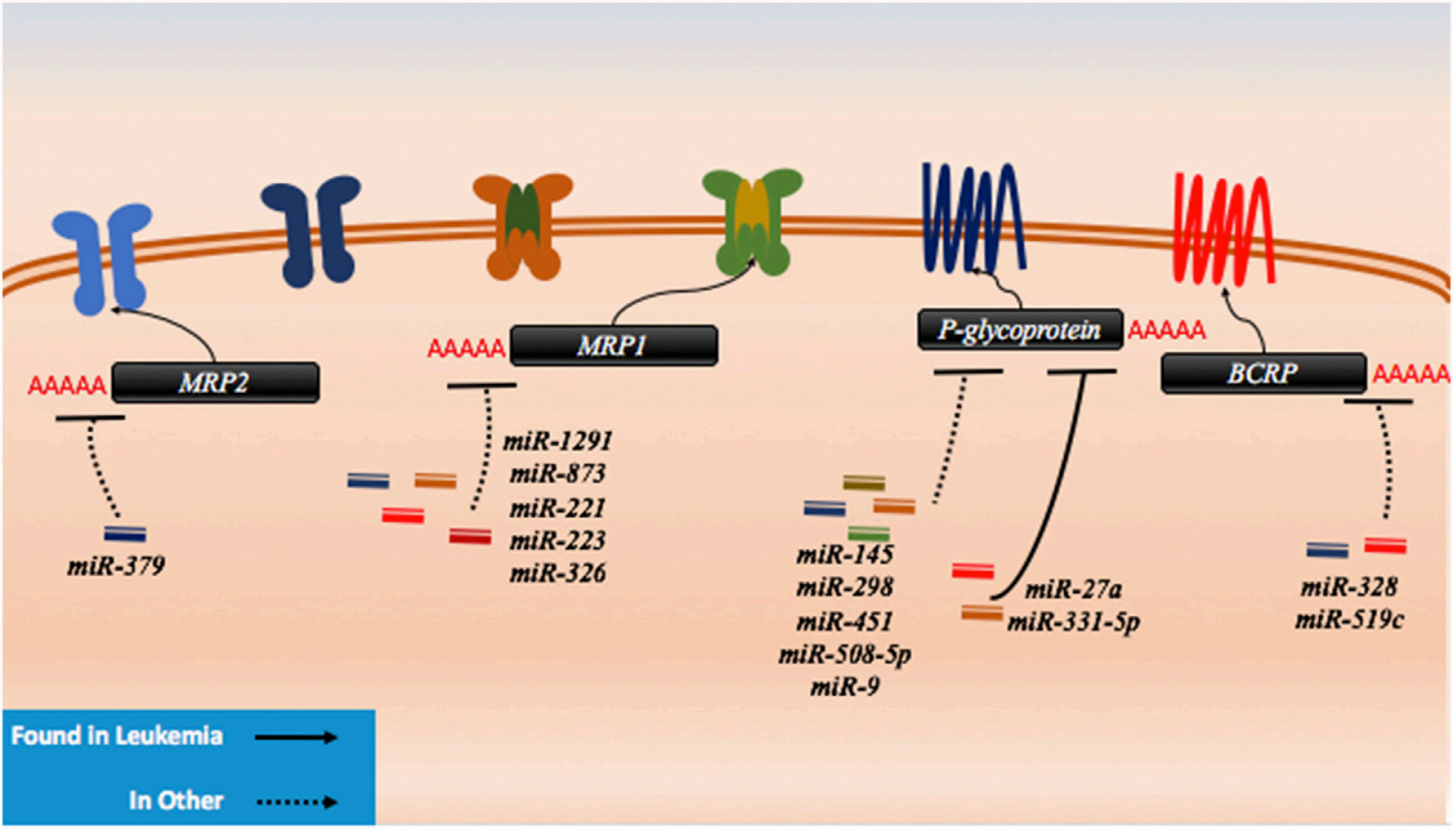
microRNAs (miRNAs) have been shown to dysregulate drug efflux mechanisms in both leukemia and other cancer. There are no known miRNA regulators of the drug influx proteins. In leukemia, P-glycoprotein has been demonstrably targeted by miR-27a and miR-331. In other cancers, P-glycoprotein has been shown to be regulated by miR-145, miR-298, miR-451, miR-508-5p, and miR-9. MRP1 has been targeted by miR-1291, miR-873, miR-221, miR-223, and miR-326, while MRP2 has been shown to be targeted my miR-379. The last protein to exhibit miRNA binding in lab setting is BCRP which has been shown to be a target of miR-328 and miR-519c.

In contrast, many efflux pumps can confer resistance to diverse and seemingly unrelated drugs and the characterization of several of these transporters has been extensive in AML. These ATP-binding cassette (ABC) proteins can be upregulated in the drug resistant forms of cancers and as such, the downregulation of miRNAs that target efflux pumps can contribute to resistance. Within this class, ABCB1 (P-glycoprotein, MDR1), ABCC1 (MRP1), ABCC2 (MRP2), and ABCG2 (BCRP) have been the most extensively examined out of 48 proteins within this functionally similar class (Figure 6) (169, 170). Indeed, previous treatments of drug resistant AML centered on the targeting of P-glycoprotein. It has been clearly demonstrated that the surface expression of P-glycoprotein is inversely proportional to the concentration of intracellular daunorubicin in blast cells and in tissue culture samples; however, blocking of P-glycoprotein did not yield positive results in clinical settings (171).

*P-glycoprotein* can be targeted by several miRNAs including *miR-27a, miR-331-5p, miR-145, miR-298, miR-508-5p, miR-9*, and *miR-451* (Figure 6; Table 5). In leukemia, only *miR-27a* and *miR-331-5p* have been demonstrated to bind to *P-glycoprotein* in the K562 and HL-60 leukemia cell lines (172). In ovarian and cervix cell lines, it was demonstrated that the downregulation of both *miR-27a* and *miR-451* can lead to downregulation of P-glycoprotein; however, in the case of *miR-27a*, this contradictory effect on P-glycoprotein is likely in part due to targeting of *HPK2* upstream (173). This was further phenotypically demonstrated by the reduced uptake of intracellular dyes and by the response to cisplatin and methotrexate (174). In more recent experiments conducted in hepatocellular carcinoma cells, in addition to direct binding to *P-glycoprotein* and *HPK2* binding, it was demonstrated that the inhibitory effect of *miR-27a* on *P-glycoprotein* may also be partially attributed to upstream modulation of the β-catenin pathway through direct binding of *SFRP1* and potentially through *FZD7* as well (172, 175). It is possible and likely that P-glycoprotein is involved in processes that are unrelated to drug trafficking as well such as apoptosis which may explain the contradictory expression in different cancers and the varying predisposition of its mutagenicity in certain cancers; however, its actions remain unclear (176).

**Table 5:** Drug trafficking gene disinhibitions caused by loss of miRNAs can lead to drug resistance.

Protein	miRNA	miRNA status in drug resistance	Sample/cancer	Mechanism	Reference
*ABCB1 (P-glycoprotein/MDR1)*	*miR-145*	Reduced	Caco2 cells, HEK293/colorectal adenocarcinoma	Reduced efflux leads to multidrug resistance	Ikemura et al. (177)

*ABCB1 (P-glycoprotein/MDR1)*	*miR-298*	Reduced	MDA-MB-231/breast cancer	Reduced efflux leads to multidrug resistance	Bao et al. (178)

*ABCB1 (P-glycoprotein/MDR1)*	*miR-27a*	Reduced	K-562, HL60, patient sample/AML A2780/ovarian cancer A2780, KB-3-1/ovarian cancer	Reduced efflux leads to multidrug resistance	Feng et al. (172) Li et al. (173) Zhu et al. (174)

*ABCB1 (P-glycoprotein/MDR1)*	*miR-331-5p*	Reduced	K-562, HL60, patient sample/AML A2780/ovarian cancer	Reduced efflux leads to multidrug resistance	Feng et al. (172)

*ABCB1 (P-glycoprotein/MDR1)*	*miR-451*	Reduced	A2780/ovarian cancer MCF-7 cells/breast cancer	Reduced efflux leads to multidrug resistance	Li et al. (173) Kovalchuk et al. (179)

*ABCB1 (P-glycoprotein/MDR1)*	*miR-508-5p*	Reduced	SGC7901/gastric cancer	Direct binding leads to reduced efflux and to multidrug resistance	Shang et al. (175)

*ABCB1 (P-glycoprotein/MDR1)*	*miR-9*	Reduced	U87 and T98G/glioblastoma multiforme	Putative or indirect knockdown. Reduced efflux leads to multidrug resistance	Munoz et al. (180)

*MRP1*	*miR-1291*	Reduced	PANC1/pancreatic cancer	Loss of binding of MRP1 contributes to doxorubicin resistance	Pan et al. (181)

*MRP1*	*miR-873*	Reduced	OVCAR3 and A2780/ovarian cancer	Loss of binding of MRP1 contributes to multidrug resistance	Wu et al. (182)

*MRP1*	*miR-221*	Reduced	NCI-H929, RPMI-8226, and U266/multiple myeloma	Loss of binding leads to MRP1-mediated drug resistance	Gullà et al. (183)

*MRP1*	*miR-222*	Reduced	NCI-H929, RPMI-8226, and U266/multiple myeloma	Loss of binding leads to MRP1-mediated drug resistance	Gullà et al. (183)

*MRP1*	*miR-326*	Reduced	MCF7/breast cancer	Inverse correlation, and likely binding of miRNA	Liang et al. (184)

*MRP2*	*miR-379*	Reduced	HepG2/hepatocellular carcinoma	Reduced miR-379 binding leads to MRP2 overexpression and increased efflux	Haenisch et al. (185)

*BCRP*	*miR-328*	Reduced	MCF7/breast cancer	Inverse correlation of the miRNA-mRNA pair, suppression of BCRP is possible and it is leading to resistance	Pan et al. (186)

*BCRP*	*miR-519c*	Reduced	S1/colon cancer	Transcript variant of BCRP loses miR-519c binding site to lead to resistance	To et al. (187)

*miRNAs, microRNAs; AML, acute myeloid leukemia*.

Direct binding of *miR-451* to *P-glycoprotein* transcripts was demonstrated in MCF-7 cells, where it was demonstrated that it could contribute to doxorubicin resistance; however, this has not yet been demonstrated to be clinically significant in cancer patients (179). In colon cancer cell-derived cell lines and HEK293 cells, it was demonstrated that *miR-145* can play a role in the repression of P-glycoprotein and increase the efflux of rhodamine 123 (177). *miR-298* was demonstrated to directly bind to the transcript in resistant breast cancer cell lines (178). This suggests that it may play a role in patients, but follow-up studies are needed. *miR-508-5p* was demonstrated to directly bind to *P-glycoprotein* in gastric cancers and its upregulation was found clinically as well (175). It has also been suggested by Munoz et al. that *miR-9* may also target P-glycoprotein and confer resistance to temozolomide in glioblastoma multiforme cells (180). These miRNAs may also prove to be relevant in AML, but no studies have been attempted to date.

While the *MRP1* gene has not demonstrated miRNA binding in AML, it was demonstrated in other cancers that the *MRP1* gene can also be targeted by miRNAs such as *miR-1291, miR-873, miR-221/222*, and *miR-326* (Figure 6; Table 5). In an analysis conducted by Pan et al., doxorubicin treatment of pancreatic cancer cells demonstrated that *miR-1291* will become upregulated and target *MRP1* directly (181). *MRP1* downregulation contributes to multidrug resistance as well in other cancers such as ovarian cancer (182). It was recently demonstrated through *in vivo* and *in vitro* studies that *miR-873* can be biologically significant in paclitaxel and cisplatin resistance in ovarian cancer cell lines where it can directly bind to *MRP1* (182). Consequently, *miR-873* is often downregulated in *MRP1*-dependent ovarian cancers. In melphalan-refractory multiple myeloma cells, Gulla et al. demonstrated that *miR-221/222* may be binding and reducing *MRP1* thus contributing to drug resistance (183). Finally, *miR-326* was inversely correlated with *MRP1* in multidrug resistant MCF7 cell lines (184). Less is known about *MRP2* targeting by miRNAs, but in the liver cell line HepG2, *miR-379* was demonstrated to be highly upregulated and to target *MRP2* directly as a response to Rifampicin resistance (185).

*BCRP*, in contrast, has been shown to be a target of *miR-520h, miR-328*, and *miR-519c* and to potentially play a role in the hematopoietic system (Figure 6; Table 5). In CD34^+^CD38^−^ hematopoietic stem cells, it was demonstrated that *miR-520h* is enriched compared to CD34^+^ cells alone and that it can directly target *BCRP* in this fraction (188). An examination of *miR-520h* in leukemic cells and AML may demonstrate a similar trend of upregulation and a contribution of *miR-520h* to drug resistance, but more experiments are required. In mitoxantrone-resistant MCF-7 cells, Pan et al. showed that the expression of *miR-328* is inversely correlated with *BCRP* and that it is directly suppressing *BCRP*, leading to resistance (186). To et al. demonstrated that *miR-519c* may play a role in downregulating BCRP in S1 colon cancer cell lines; however, they demonstrated that binding of *miR-519c* was limited to a longer form of the transcript only found in their parental cell line compared to their mitoxantrone-resistant counterpart (187, 189). This study highlights the importance of splice variants and how they may gain or lose miRNA-binding sites and thereby contribute to resistance.

## Implications in Treatment

Drug resistance is only a single aspect of clinical setbacks; however, it is a major contributor to therapy failure. Although treatment has improved substantially in some cancers in the past few decades, many other cancer types continue to demonstrate substantial patient populations that relapse after an initially successful treatment. While we focused on the regulation of drug resistance-associated miRNAs common between different cancers and drug classes, there are likely various miRNA that are specific to different drug treatments and cancers. However, the miRNA dysregulations discussed may have therapeutic value beyond AML. Furthermore, although we describe several drug resistance proteins, our analysis only focused on miRNA specifically implied in drug resistance where they were demonstrated to have direct activity and as such, the list is not exhaustive (190).

There are also many other molecular changes that occur in the development of drug resistance such as copy number variations, aberrant methylation, and aberrant post-transcriptional and post-translational processing (191, 192). The modulation of miRNAs offers a new perspective on drug resistance as miRNA replacement therapy and miRNA inhibition therapy raises the potential of developing new and effective drug therapies. Subtle miRNA changes can lead to significant changes in protein-coding gene expression and can consequently lead to changes in tumor progression and patient outcome. Experimental success *in vitro* and *in vivo* models may point to the likely coming of more miRNA-based clinical trials.

Previously, Mrx34 emerged as a promising therapy for the treatment of unresectable primary liver cancer. Due to multiple immune-related adverse events, this therapy was terminated in phase I although there was evidence of benefit in a subset of patients (193). Its promise came from being a p53-response element that was thought to mediate p53’s antitumor effects and consequently affecting downstream signaling in proliferation arrest and induction of apoptosis by targeting *c-MYC, CDK6*, and *c-MET* (194). However, recent research now demonstrates that it may not always behave as a tumor-suppressor either and furthermore, p53 may also be a direct target of *miR-34a* (138, 195). In liver cancers with β-catenin mutations, it is demonstrated that LNA-34a, a *miR-34a* inhibitor, displays antitumor effects. This is suggested to occur through blocking HNF-4α targeting which in turn decreases cyclin D1 and inhibits proliferation (196, 197).

A *miR-16* mimic has also been recently introduced in patients in an open-label phase I clinical trial for mesothelioma and non-small cell lung cancer (NSLC). *miR-16* was shown to be dysregulated in many different cancers (87, 89, 90, 103–105, 124, 198). A directed analysis in mesothelioma showed that *miR-16* is reduced in patient samples and that a knock-in of a *miR-16* mimic is tumor suppressive (198). This observation was repeated in xenografted mice with high success (198). Currently, there are no miRNA-based therapies for drug resistant AML or AML-related diseases.

Currently, there are two miRNA-based therapies intended to treat different cancers that are on-going or with pending results. MesomiR-1, a *miR-16* mimic, was in a multi-center Phase I trial intended to treat mesothelioma and NSLC. This trial has been completed as of January 2017 and the results are currently pending. MRG-106 is a miRNA inhibitor that targets *miR-155* that is currently being examined in cutaneous T-cell lymphoma and mycosis fungoides. Like mesomiR-1, it is also currently in phase I. It is thought to block the action of *miR-155* from targeting tumor suppressors such as *C/EBP*β and altering the TGF-β response (199). This study is currently still recruiting patients. These studies may offer promise of miRNA treatment as therapy and pave the way for future studies similar in nature.

## Concluding Statement

Today, the main hurdle for miRNA-based therapies remains to be the method of delivery. Many types of viruses are thought to be potentially useful for treatment and many stabilizing modifications such as phosphorothioate, methyl- and fluoro-substitutions on RNA species may help to overcome this hurdle (200, 201). Given the diverse set of roles that miRNAs play in regular cellular function, it is evident that clear elucidation of specific miRNA mechanisms may be required before their integration into modern cancer therapy (202). In contrast, due to the dependence and overexpression of a few coding mRNA in tumorigenic cells, it is possible that miRNAs may have a higher therapeutic index. miRNAs may prove to be an important addition to treatment in the years to come to treat drug resistant cancers in the future.

## Author Contributions

MG contributed to the research, figure design, and writing of manuscript. LS contributed to the research, editing, and overall design of manuscript.

## Conflict of Interest Statement

The authors declare that the research was conducted in the absence of any commercial or financial relationships that could be construed as a potential conflict of interest.
